# Alcohol-related disorders and associated factors in a rural area in Brazil

**DOI:** 10.11606/S1518-8787.2018052000262

**Published:** 2018-09-13

**Authors:** Gustavo Pêgas Jaeger, Christian Loret de Mola, Mariangela Freitas Silveira

**Affiliations:** I Universidade Federal de Pelotas. Faculdade de Medicina. Programa de Pós-Graduação em Epidemiologia. Pelotas, RS, Brasil; II Universidade Federal de Pelotas. Faculdade de Enfermagem. Departamento de Enfermagem. Pelotas, RS, Brasil; III Universidade Federal de Pelotas. Faculdade de Medicina. Departamento Materno Infantil e de Pós-Graduação em Epidemiologia. Pelotas, RS, Brasil

**Keywords:** Alcohol Drinking, epidemiology, Alcohol-Induced Disorders, epidemiology, Alcoholism, epidemiology, Risk Factors, Socioeconomic Factors, Rural Population, Consumo de Bebidas Alcoólicas, epidemiologia, Transtornos Induzidos por Álcool, epidemiologia, Alcoolismo, epidemiologia, Fatores de Risco, Fatores Socioeconômicos, População Rural

## Abstract

**OBJECTIVE:**

To describe the prevalence of alcohol-related disorders and associated factors in an exclusively rural population.

**METHODS:**

This is a cross-sectional, population-based study of a rural research consortium, conducted in a medium-sized city in Southern Brazil, with adults living in a rural area, using the AUDIT (Alcohol Use Disorders Identification Test). The analysis included the prevalence of alcohol-related disorders and associated factors, such as the sociodemographic, family, and health factors; it was carried out by Poisson regression, in a hierarchical analysis model, with a 95% confidence interval.

**RESULTS:**

The final sample amounted to 1,519 subjects. The prevalence of alcohol-related disorders (AUDIT ≥ 8) was 8.4% (95%CI 7.0–9.8). Risk factors for alcohol-related disorders were being male (PR = 8.2, 95%CI 4.82–14.16), age group between 18 and 29 years (PR = 3.29, 95%CI 1.80–6.0), and smoking (PR = 1.88, 95%CI 1.03–3.43). The practice of religion (PR = 0.38, 95%CI 0.25–0.58) and education level between nine and 11 years (PR = 0.33, 95%CI 0.16–0.69) were protective factors with statistical significance. Marital status and social status were not associated with the outcome studied.

**CONCLUSIONS:**

The prevalence of alcohol-related disorders in the rural population is high, but, on average, it is lower than that found in urban populations. Risk and protective factors were similar to those found in previous studies. Men, younger persons, and smokers are at higher risk for alcohol-related disorders. On the other hand, practicing a religion and having a higher education level were protective factors.

## RESUMO

**OBJETIVO:**

Descrever a prevalência de transtornos relacionados ao uso de álcool e fatores associados em uma população exclusivamente rural.

**MÉTODOS:**

Estudo transversal de base populacional, integrante de um consórcio de pesquisa rural, realizado em cidade de médio porte do Sul do Brasil, com adultos residentes na zona rural, utilizando o AUDIT (*Alcohol Use Disorders Identification Test*). A análise incluiu a prevalência dos transtornos relacionados ao uso de álcool e fatores associados, como sociodemográficos, familiares e de saúde; foi realizada por regressão de Poisson, em modelo hierarquizado de análise, com intervalo de confiança de 95%.

**RESULTADOS:**

A amostra final foi de 1.519 indivíduos. A prevalência de transtornos relacionados ao uso de álcool (AUDIT ≥ 8) foi de 8,4% (IC95% 7,0–9,8). Os fatores de risco para transtornos relacionados ao uso de álcool foram sexo masculino (RP = 8,26; IC95% 4,82–14,16), faixa etária entre 18 e 29 anos (RP = 3,29; IC95% 1,80–6,0) e tabagismo (RP = 1,88; IC95% 1,03–3,43). Como fatores de proteção com significância estatística encontramos a prática de religião (RP = 0,38; IC95% 0,25–0,58) e escolaridade entre nove e 11 anos de estudo, (RP = 0,33; IC95% 0,16–0,69). Estado civil e classe social não estiveram associados com o desfecho estudado.

**CONCLUSÕES:**

A prevalência de transtornos relacionados ao uso de álcool na população rural é alta, porém, em média, mais baixa do que as encontradas em populações urbanas. Os fatores de risco e proteção foram similares aos encontrados em estudos prévios. Os homens, as pessoas mais jovens e os tabagistas têm maior risco para apresentar transtornos relacionados ao uso de álcool. Por outro lado, praticar uma religião e ter maior escolaridade foram fatores de proteção.

## INTRODUCTION

The pattern of alcohol consumption of the adult Brazilian population has been the object of several research studies in the last decades^1–3^. These studies are important because this pattern is related to several health problems, as well as social and economic issues^2^ ^,^ ^4^.

Current scientific literature uses different nomenclatures to refer to different patterns of alcohol consumption^5^. The World Health Organization (WHO) defines three for them. The at-risk use is described as a pattern that increases the possibility of harm to the consumer or others. Harmful use is defined as a pattern that results in physical or mental harm. Dependence is characterized by a set of physiological, behavioral, and cognitive signs and symptoms, such as a strong desire to consume alcohol and difficulty to stop consumption, which denote a more serious pattern^5^.

In a report published by the WHO in 2014, alcohol is described as a substance capable of leading to chemical dependence and causing serious health problems, such as liver cirrhosis, various cancers, and pancreatitis^4^. The number of deaths attributed to alcohol consumption in 2012 was 3.3 million, representing 5.9% of all deaths in the world that year^4^. Harmful alcohol consumption leads to economic losses to nations. South Korea and Thailand, middle-income countries as Brazil, had a total cost with alcohol abuse of approximately 2.1% of their gross domestic product^6^. In Brazil, large national studies have researched alcohol consumption. The II National Survey of Alcohol and Drugs (LENAD-II) carried out in 2012 found an overall prevalence of alcohol dependence of 6.8%^2^. Another large survey, the National Health Survey (PNS) of 2013, found a prevalence of 10.3% of alcohol abuse, defined as the consumption of five or more doses for men and four or more doses for women on a single occasion in the last 30 days^3^. In addition to these national surveys, smaller studies have been carried out to know in detail the pattern, prevalence, and factors associated with alcohol consumption in Brazil. A study conducted in 2012^7^ with the urban population of the city of Florianópolis, State of Santa Catarina, Brazil, using the Alcohol Use Disorders Identification Test (AUDIT) questionnaire^5^, found a prevalence of 18.4% of alcohol-related disorders. In Pelotas, State of Rio Grande do Sul, Brazil, in 2004, a study also conducted with the urban population found a prevalence of 14.3% of alcohol abuse (30 grams or more per day)^8^.

Some factors associated with higher alcohol consumption are known and more consistent in current literature, such as being male, being single or divorced, and being a smoker. However, the relationship with some important sociodemographic factors, such as age group, social class, education level, income, among others, is not well established^1,7–9^.

Although knowledge about the pattern of alcohol consumption in the Brazilian population has grown considerably, few data can be found in the literature on it in rural populations^2^ ^,^ ^3^ ^,^ ^10^. In one of these studies with *quilombola* communities, in 2015, a prevalence of 10.7% was found for excessive alcohol consumption and an association with higher education level, smoking, and work exercise. Excessive consumption in this study was classified as five or more doses for men and four or more doses for women on a single occasion or regular consumption of 15 or more doses per week^10^.

Unlike Brazil, we can find studies in the literature in other countries on alcohol consumption and alcohol-related problems in exclusively rural regions. A study conducted in rural India in 2013 found a prevalence of 9.4% of alcohol consumption and 3.7% of at-risk alcohol consumption. In this study, alcohol consumption was associated with males, intermediary age group (15–44 years), low education level, and smoking^11^. Another study carried out with the rural population of Vietnam in the same year found a prevalence of alcohol-related problems of 11.8%, with a strong association with males^12^.

In this study, we describe the prevalence of alcohol-related disorders and their associated factors in the rural population of a medium-sized city in Southern Brazil.

## METHODS

This population-based, cross-sectional study was conducted in the rural area of the city of Pelotas, State of Rio Grande do Sul, Brazil, with individuals aged 18 years or over. It is part of the research consortium of the 2015/2016 biennium of the Graduate Program in Epidemiology of the Universidade Federal de Pelotas^13^. Pelotas is located in the Southern region of the state of Rio Grande do Sul. The total population of the municipality in 2010 was 328,275 inhabitants, of which approximately 17,000 were aged 18 years or over and lived in the rural area. The rural area of Pelotas consists of eight districts, divided in 50 census tracts according to criteria of the Brazilian Institute of Geography and Statistics (IBGE)^14^.

The sample consisted of individuals from all districts of the rural area of the city of Pelotas. The sample size of the consortium was defined based on the needs of nine subprojects. For this study, the minimum sample size was 1,440 individuals, calculated in the OpenEpi program^15^. The parameters used to calculate the sample size were 15% prevalence of the main outcome (alcohol-related disorders), estimated by the mean prevalence of two studies with similar design and another one performed in the urban area of Pelotas^7^ ^,^ ^8^ ^,^ ^16^, acceptable error of three percentage points (pp), design effect of two, 95% confidence interval, statistical power of 80%, minimum odds ratio of 1.8, in addition to 10% for losses and refusals and 15% for confounding factors.

The sampling process was carried out in multiple stages. Initially, 24 of the 50 census tracts that make up the rural area were drawn. Then, 30 households from each tract were randomly selected to reach the minimum sample value, estimating that each household would have at least two adults. We used the Google Earth software to identify households in each tract.

Field work was carried out between January and June 2016. A group of interviewers was trained to apply the research instruments, using electronic questionnaires on tablets, using RedCap (Research Electronic Data Capture) software, and traditional paper questionnaires. They also received training and were standardized in the collection of anthropometric data. The field work always had the supervision of at least two Master’s students responsible for the consortium.

In the field of research, it was fundamental to use a GPS device (Global Positioning System). From a previously defined process for sample selection, the path started using the geographical coordinates provided by the GPS for the location of the residences. Further details on the methodology of the field work are available in the methodological article of this research consortium^16^.

To evaluate alcohol consumption, we used the AUDIT, which is a screening instrument developed by the WHO, already validated in Brazil^7^ ^,^ ^17^, with 10 questions that can generate a total score from zero to 40 points. In this study, we used the AUDIT version validated in the city of Pelotas^17^. The WHO proposes a subdivision, according to the scores obtained in the AUDIT, into four categories or patterns of alcohol consumption: zero to seven, low-risk use; eight to 15, at-risk use; 16 to 19, harmful use; and 20 or more, likely dependent. In addition, the AUDIT is also used in a dichotomized manner, from zero to seven points in the category considered “low-risk use”, and ≥ 8 points in the category called “alcohol-related disorders”, used as the outcome of this study, which includes the subcategories at-risk use, harmful use, and likely dependent^7^.

The exposure variables studied were categorized as: sex (male; female), age group (18–29; 30–39; 40–49; 50–59; ≥ 60 years), self-reported race (white; black; brown, yellow, or indigenous), self-reported family ancestry (German or Pomeranian; Brazilian or mixed; Italian; Portuguese; African or *Quilombola;* Polish; other), family history of alcohol-related problems (no; yes), education level in full years (zero; 1–4; 5–8; 9–11; ≥ 12), marital status (married or with partner; divorced, separated, or widowed; single), current work (no; yes), socioeconomic class (A–B; C; D–E), using the Brazilian Association of Research Companies (ABEP)^18^, religion (no; yes), religious choice (Catholic; Evangelical; Afro-Brazilian; Spiritist; other), religious practice in the last 30 days (none; ≥ once), using the question “From <day> of last month, how often did you go to Mass, worship, or religious session?”, smoking (current smoker; former smoker; never smoked), likely current diagnosis of depression (no; yes), using the Edinburgh depression scale with cutoff point ≥ 13 for likely diagnosis^19^, alcohol use at some point in life (no; yes), age of first experience with alcohol (< 18; ≥ 18 years).

In order to perform the bivariate and multivariate association analyses, we chose to use the dichotomized version of the outcome variable to increase the statistical power. In these analyses, we used the Wald test for heterogeneity and trend test to calculate p values. We performed multivariate analyses using a hierarchical conceptual and theoretical model^20^ at four levels, from the most distant to the most proximal to the outcome.

At the first level of the model, we include the variables of sex, age, family ancestry, and family history of alcoholism. In the second level, we included marital status, education level, current work, socioeconomic classification, and type of religion. The third level consisted of only the variable of religious practice. In the fourth and last hierarchical level, we included the variables of smoking, likely diagnosis of depression, and age of first experience with alcohol.

The hierarchical model was used as the basis for the statistical analyses. The variables of each hierarchical level were adjusted to each other. From the second level, the adjustments were also made by the variable clusters of the previous levels. This approach presents some advantages, such as keeping the adjustment for potentially confounding variables and avoiding adjustment for possibly mediating variables of the studied process^20^.

The analyses were performed by Poisson regression for the calculation of prevalence ratios (PR), and we kept the variables with p < 0.20 in the bivariate analysis in the model. Throughout the statistical analysis process, we used the *svy* command because the sampling process was carried out in multiple stages, with drawing of clusters and not individuals, in addition to considering the different sample sizes in each district. We performed the analyses using the Stata 14.1 statistical package.

Quality control was carried out by applying 10 questions from the original questionnaire to 10% of the total sample by telephone. Concordance was calculated by the Kappa test.

The study was approved by the Research Ethics Committee of the Faculdade de Medicina of the Universidade Federal de Pelotas (Process 1.363.979, December 11, 2015). All research participants signed an informed consent.

## RESULTS

The sample consisted of 1,697 individuals living in the rural area. Of these, 1,519 completed the research, which resulted in 10.5% of losses and refusals. The weighted Kappa coefficient was 0.90 for the smoking question and 0.51 for the question on the age of the first experience with alcohol. Of the sample, 51.7% were female, 85.2% self-reported as whites, and 41.0% self-reported as descended from Germans or Pomeranians. Most had five to eight years of study (42.6%), worked at the time of the research (59.0%), and belonged to socioeconomic class C (53.7%). Regarding marital status, 60.3% reported being married or living with a partner, and 4.4% reported being divorced or separated. Most (86%) reported having some kind of religion. Of these, 46.5% self-reported as Catholics and 45.0% as Evangelicals. In addition, 50.6% reported practicing their religion at least once a month (Table 1).

**Table 1:** Description of the sample of individuals aged 18 years or over living in the rural area of the city of Pelotas, State of Rio Grande do Sul, Brazil, 2016. (n = 1,519)

Characteristic	n^a^	%
Sex		
Male	734	48.3
Female	785	51.7
Age group (years)		
18–29	287	18.9
30–39	228	15.0
40–49	296	19.5
50–59	297	19.5
60 or over	411	27.1
Race		
White	1,296	85.0
Black	92	6.0
Brown	101	6.9
Yellow	21	1.4
Indigenous	9	0.7
Family ancestry		
German/Pomeranian	632	41.0
Brazilian/Mixed	386	26.0
Italian	171	11.4
Portuguese	88	5.8
African/*Quilombo*	75	5.0
Other	167	10.8
Family history of problems with alcohol		
No	1,074	70.9
Yes	441	29.1
Education level		
0	39	2.6
1–4	435	28.7
5–8	648	42.6
9–11	360	23.6
12 or over	37	2.5
Marital status		
Married/Partner	920	60.3
Divorced/Separated	67	4.4
Single	397	26.4
Widow	135	8.9
Current work		
No	613	41.0
Yes	906	59.0
Socioeconomic class (ABEP)		
A or B	301	20.0
C	814	53.7
D or E	388	26.3
Religion		
No	212	14.0
Yes	1,307	86.0
Religious choice		
Evangelical	595	45.0
Catholic	599	46.5
Spiritist	38	2.9
Afro-Brazilian	11	0.9
Other	61	4.7
Religious practice		
Zero/month	660	49.4
≥ 1/month	645	50.6
Smoking		
Never smoked	987	64.7
Former smoker	285	18.7
Current smoker	247	16.6
Diagnosis of depression^b^		
No	1,287	89.0
Yes	155	11.0
Use of alcohol		
Never	169	11.1
At least once	1,350	88.9
Age of first experience with alcohol (years)		
< 18	853	64.9
≥ 18	458	35.1

ABEP: *Associação Brasileira de Empresas de Pesquisa* (Brazilian Association of Research Companies)

^a^ Different sample values because of missing data.

^b^ Edinburgh postnatal depression scale – cutoff point ≥ 13.

**Figure 1:**
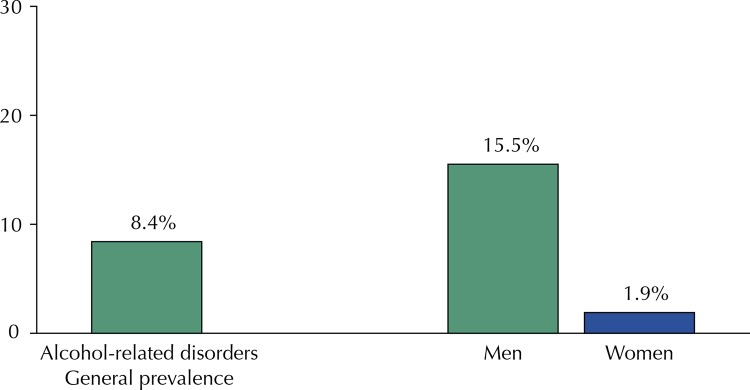
Prevalence of alcohol-related disorders (AUDIT ≥ 8) in adults living in the rural area of Pelotas, State of Rio Grande do Sul, Brazil, 2016.

**Figure 2:**
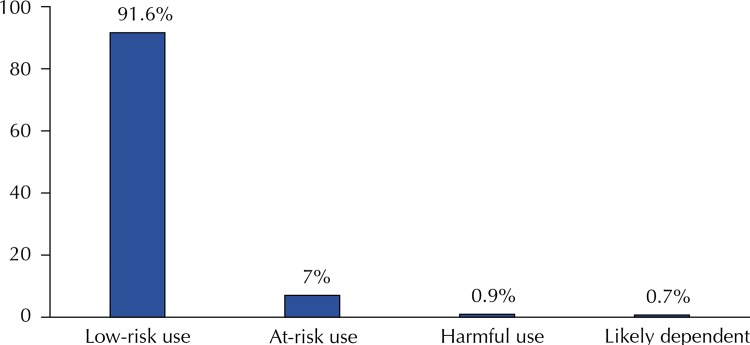
Categories of pattern of alcohol consumption in adults living in the rural area of Pelotas, State of Rio Grande do Sul, Brazil, 2016.

We found alcohol-related disorders, an AUDIT score category ≥ 8 points, in 8.4% of the 1,519 adults studied, with a significant difference between sexes, being it 15.5% in men and 1.9% in women. Among the patterns of alcohol consumption, likely dependence presented a prevalence of 0.7% (Figures 1 and 2).

In the multivariable analysis, the risk of alcohol-related disorders, the AUDIT category of outcome used, was higher in males (PR = 8.26, 95%CI 4.82–14.16). The age group of 18–29 years presented a prevalence 3.3 times higher (95%CI 1.80–6.00) than the category of 60 years or over. In addition, the other categories also presented higher prevalences in relation to the reference group, but they tended to decrease with increasing age (p < 0.001) (Table 2).

**Table 2:** Multivariate analysis of alcohol-related disorders (AUDIT ≥ 8) and exposure variables. Persons aged 18 years or over living in the rural area of the city of Pelotas, State of Rio Grande do Sul, Brazil, 2016.

Variable	Unadjusted analysis		Adjusted analysis	
	
	n (%)	PR (95%CI)	n (%)	PR (95%CI)
1st level^c^				

Sex		p < 0.001^a^		p < 0.001^a^
Male	112 (15.3)	8.27 (4.80–14.24)	111 (15.2)	8.26 (4.82–14.16)
Female	15 (1.9)	1	15 (1.9)	1
Age groups (years)^a^		p < 0.001^b^		p = 0.001^b^
18–29	34 (11.9)	3.57 (1.94–6.56)	34 (11.9)	3.29 (1.8–6.0)
30–39	24 (10.5)	3.24 (1.60–6.55)	24 (10.5)	2.94 (1.42–6.13)
40–49	27 (9.2)	2.80 (1.57–4.89)	27 (9.2)	2.56 (1.39–4.68)
50–59	29 (9.7)	3.04 (1.61–5.71)	28 (9.4)	2.86 (1.53–5.34)
≥ 60	13 (3.2)	1	13 (3.2)	1
Race		p = 0.224^a^		p = 0.145^a^
Other	2 (6.7)	0.96 (0.23–3.98)	2 (6.7)	0.76 (0.19–2.94)
Black/Brown	22 (11.4)	1.46 (0.94–2.25)	22 (11.5)	1.43 (1.00–2.05)
White	103 (8.0)	1	102 (7.9)	1
Family history		p = 0.184^a^		p = 0.067^a^
No	82 (7.6)	0.76 (0.51–1.14)	82 (7.6)	0.72 (0.51–1.02)
Yes	44 (10.0)	1	44 (10.0)	1

2nd level^d^				

Education level (full years)		p = 0.108^a^		p = 0.013^a^
0–4	30 (6.3)	0.41 (0.15–1.10)	30 (6.3)	0.42 (0.18–1.01)
5–8	64 (9.9)	0.64 (0.26–1.57)	64 (9.9)	0.57 (0.26–1.24)
9–11	27 (7.5)	0.46 (0.21–1.00)	27 (7.5)	0.33 (0.16–0.69)
≥ 12	6 (16.2)	1	6 (16.2)	1
Marital status		p = 0.018^a^		p = 0.273^a^
Single	55 (13.9)	2.02 (1.24–3.29)	55 (13.9)	1.41 (0.82–2.44)
Divorced/Widower/Separated	10 (4.9)	0.73 (0.40–1.30)	10 (5.0)	1.32 (0.70–2.52)
Current work		p < 0.001^a^		p = 0.114^a^
No	29 (4.7)	0.41 (0.28–0.60)	29 (4.7)	0.69 (0.44–1.10)
Yes	98 (11.0)	1	98 (11.0)	1
ABEP		p = 0.190^a^		p = 0.192^a^
A or B	30 (9.6)	0.89 (0.49–1.60)	30 (9.6)	0.88 (0.50–1.53)
C	58 (6.8)	0.64 (0.40–1.04)	58 (6.8)	0.66 (0.42–1.03)
D or E	35 (10.4)	1	34 (10.1)	1
Religious choice		p = 0.008^a^		p = 0.09^a^
Evangelical	33 (5.6)	0.46 (0.27–0.78)	33 (5.6)	0.70 (0.38–1.30)
Other	8 (7.3)	0.60 (0.27–1.32)	8 (7.3)	0.92 (0.45–1.85)
Catholic	61 (10.2)	0.85 (0.54–1.32)	61 (10.2)	1.25 (0.79–1.98)
No religion	25 (11.8)	1	24 (11.4)	1

3rd level^e^				

Religious practice		p < 0.001^a^		p = 0.02^a^
Zero/month	73 (11.4)	2.55 (1.72–3.78)	73 (11.4)	1.62 (1.08–2.43)
≥ 1/month	29 (4.4)	1	29 (4.4)	1

4th level^f^				

Smoking		p < 0.001^b^		p = 0.033^b^
Smoker	43 (17.4)	3.00 (1.87–4.81)	31 (17.4)	1.88 (1.03–3.43)
Former smoker	28 (9.8)	1.69 (1.16–2.46)	25 (11.4)	1.29 (0.81–2.05)
Never smoked	56 (5.7)	1	44 (6.4)	1
Diagnosis of depression		p = 0.396^a^		p = 0.814^a^
No	11 (7.0)	0.75 (0.39–1.46)	6 (5.8)	0.90 (0.39–2.10)
Yes	114 (8.8)	1	94 (9.6)	1
Age of first experience with alcohol (years)		p = 0.012^a^		p = 0.189^a^
< 18	97 (11.4)	1.66 (1.12–2.46)	75 (10.7)	1.31 (0.86–2.00)
≥ 18	30 (6.5)	1	25 (6.5)	1

ABEP: *Associação Brasileira de Empresas de Pesquisa* (Brazilian Association of Research Companies)

^a^ P-value of the test of heterogeneity.

^b^ P-value of the linear trend.

^c^ Adjustment for all variables of the first level.

^d^ Adjustment for the variables of the 1st level with p < 0.20 and for all variables of the 2nd level.

^e^ Adjustment for upper level variables with p < 0.20 and for religious practice.

^f^ Adjustment for upper level variables with p < 0.20 and for variables of the level.

In the category of education level, those who completed nine to 11 years of school presented a lower prevalence of the outcome than the reference category, i.e., those with 12 years or more of study (PR = 0.33, 95%CI 0.16–0.69). The other categories also presented lower prevalences, but without statistical significance.

Individuals who self-reported as Evangelicals had a lower prevalence of outcome than those without religion (PR = 0.70, 95%CI 0.38–1.30), but the association was not statistically significant. Religious practice one or more times in the last 30 days was also a protective factor for the presence of alcohol-related disorders. Those who had no religious practice in the last 30 days had a 1.6 times higher prevalence (95%CI 1.08–2.43) (Table 2).

We also found a relation between smoking and alcohol-related disorders. After the adjusted analysis, current smokers had a 1.9 times higher prevalence of alcohol-related disorders (95%CI 1.03–3.43) than those who had never smoked (Table 2).

## DISCUSSION

The prevalence of alcohol-related disorders (AUDIT ≥ 8) found in this study was 8.4%. We found higher prevalences among males, the 18–29 age group, those who did not practice their religion, and smokers.

We found no association between the outcome variable, i.e., alcohol-related disorders, and the following exposure variables: marital status, family history of alcohol problems, social class, religious choice, likely diagnosis of depression, and age of first experience with alcohol.

Overall, the prevalence of 8.4% of alcohol-related disorders was lower than those found in many studies on the subject^8,11,12,21–23^. For example, in rural regions in India, the prevalence of at-risk alcohol consumption (AUDIT ≥ 8) was 33.2%^24^.

Prevalence findings, however, are similar to those found in other studies. The PNS, for example, has found a prevalence of alcohol abuse of 10.3% in adult individuals living in rural areas of Brazil^3^. In predominantly rural *quilombola* communities in Bahia, the prevalence of excessive alcohol consumption was 10.7%^10^. In 2013, in India, a study using the same instrument and the same cutoff point as we used found a prevalence of 9.4%^11^. Another study carried out in a rural region of Kenya has found a prevalence of 7.7% of alcohol-related disorders, using the AUDIT questionnaire and cutoff point ≥ 8^25^.

To compare the different prevalences found in some studies, it is important to take into account the measurement methods used, such as the questionnaire and cutoff point, for example. Two studies conducted in Brazil, in the cities of Florianópolis, State of Santa Catarina, and Rio Grande, State of Rio Grande do Sul, have used the AUDIT questionnaire with the same cutoff point as this study^7^ ^,^ ^21^, which allows us to better compare the findings. The prevalence found in this study is very close to that found in Rio Grande, of 7.9%, a city close to Pelotas and with very similar characteristics, and it is different from the prevalence of 18.4% found in Florianópolis. This result may indicate that the characteristics of different regions and populations studied, such as the number of inhabitants and cultural habits, may be related to different patterns of consumption.

Particularly in Brazil, there are few studies conducted exclusively with rural populations, which limits comparability with the results found. With the data currently available, we can see that the prevalence found in this study in the rural area of Pelotas was lower than that identified in the urban area of this city^8^, which may be due to differences in the methods used in both studies. Two other large studies evaluating all regions of the country have also found lower prevalences of at-risk consumption in rural areas. These results may suggest that at-risk alcohol consumption is lower in rural populations than in urban populations^2^ ^,^ ^3^ ^,^ ^8^.

The factor most strongly associated with alcohol-related disorders in this study was being male, which corroborates almost all studies on the subject^8^ ^,^ ^9^ ^,^ ^21^. The literature has shown that men drink more than women, which may partially explain the higher prevalence of at-risk consumption in this group^2^ ^,^ ^3^. Although there is no solid explanation for this association, the complex combination of genetic, behavioral, and cultural factors for men may play an important role^26^.

Regarding age group, the main risk factor belonged to the group between 18–29 years, as shown by most studies^9^ ^,^ ^23^. Some factors could act as a risk for younger persons, such as the transitional phase of life (entry into adult life), higher frequency of personal and family conflicts, and greater susceptibility to advertising campaigns that encourage alcohol consumption^23^ ^,^ ^27^. It is important to point out, for a better interpretation of this result, that the method used in this study, the score in AUDIT ≥ 8, allows us to detect from lighter patterns of consumption to more severe patterns. Higher prevalences in younger age groups may be due to the higher number of individuals with milder patterns of at-risk consumption, but which are already considered, by the classification adopted, as alcohol-related disorders.

This study did not find a statistically significant association between marital status and alcohol-related disorders. However, single individuals had higher prevalences than those married or living with a partner^7^ ^,^ ^28^. One possible explanation for this relationship may be that individuals in more stable relationships have better health-related habits^29^. The size of the sample studied may not have been enough to find a statistically significant association.

Regarding religious choice, although the association was not statistically significant, we found results that are similar to other studies on the subject. Individuals who claim to be Evangelical had lower prevalences of alcohol-related disorders^23^. In addition, individuals who practice their religions, that is, attend one or more times a month places of practice, presented lower prevalences of the outcome studied. This association is likely to be related to a broader context of support and social support. We can also understand this association by thinking about the guidance of each religion (some with a greater emphasis on healthy behaviors and habits, such as abstinence from alcohol) and how adepts follow it^23^.

This study also found a positive association between smoking and alcohol-related disorders. Smokers presented higher prevalences than former smokers and those who never smoked, as already found in the current literature^8^ ^,^ ^11^ ^,^ ^21^. The explanation for this double addiction may lie in the complex genetic-environmental interaction, possibly from a common cause of the two behaviors, be it genetic characteristics predisposing to addiction, environmental exposure to chemicals consumed by relatives, or both conditions^30^.

The great differential of this research is that it is a population-based study with an exclusively rural population. However, some limitations are relevant and need to be highlighted and discussed. Our sampling process may have reduced the participation of very distant households in the research^16^. However, even though it is a limitation, we believe that it has not been able to distort the results found. Another condition that should be thought of as a limitation is the way how alcohol consumption was researched. The questionnaire was applied with interviews and not self-administered questionnaires, which may have led some individuals to minimize their pattern of consumption. This limitation may have led to an underestimation of the prevalence of alcohol-related disorders. However, we believe that the truly underestimated value was the most severe patterns of consumption, with AUDIT score ≥ 20.

Other possible limitations come from the study having a cross-sectional design. Causal inferences are limited, which demands a careful interpretation of the data. In addition, there is the possibility of reverse causality in some associations, such as marital status, smoking, and religious choice and practice. We believe that association with religious practice should be better understood in future studies, with designs more suitable for it. It is possible that current abstainers have sought religion as a way to help them with their alcohol-related problems.

The generalization of the results of this research is probably limited, since we studied a specific rural population. Rural regions of different places in Brazil may have peculiar characteristics in relation to different habits, such as the consumption of alcohol. However, we believe that this study is an improvement in the knowledge on less studied populations, such as rural ones, and it is a possible motivator for further research with populations living in rural areas of other regions in Brazil.

The prevalence of alcohol-related disorders in the rural population studied is high, but it is lower than those found by other studies in the country, mostly in urban populations. The factors associated with alcohol-related disorders found are similar to those identified in other studies. This knowledge from repeated findings contributes to the adoption of public health measures at different levels of prevention. However, there is a clear lack of data on alcohol consumption in rural populations in Brazil. More studies need to be carried out with rural populations in order to further the knowledge on their characteristics and needs.
